# Improving the healthcare response to domestic violence and abuse in UK primary care: interrupted time series evaluation of a system-level training and support programme

**DOI:** 10.1186/s12916-020-1506-3

**Published:** 2020-03-05

**Authors:** Alex Hardip Sohal, Gene Feder, Kambiz Boomla, Anna Dowrick, Richard Hooper, Annie Howell, Medina Johnson, Natalia Lewis, Clare Robinson, Sandra Eldridge, Chris Griffiths

**Affiliations:** 1 Institute of Population Health Sciences, Queen Mary University of London, London, UK; 2 Centre for Academic Primary Care, Population Health Sciences, Bristol Medical School, University of Bristol, Bristol, UK; 3 IRISi, Bristol, England

**Keywords:** Domestic violence abuse, Complex, Evaluation, Improvement, Implementation, Interrupted time-series, Observational

## Abstract

**Background:**

It is unknown whether interventions known to improve the healthcare response to domestic violence and abuse (DVA)—a global health concern—are effective outside of a trial.

**Methods:**

An observational interrupted time series study in general practice. All registered women aged 16 and above were eligible for inclusion. In four implementation boroughs’ general practices, there was face-to-face, practice-based, clinically relevant DVA training, a prompt in the electronic medical record, reminding clinicians to consider DVA, a simple referral pathway to a named advocate, ensuring direct access for women to specialist services, overseen by a national, health-focused DVA organisation, fostering best practice. The fifth comparator borough had only a session delivered by a local DVA specialist agency at community venues conveying information to clinicians. The primary outcome was the daily number of referrals received by DVA workers per 1000 women registered in a general practice, from 205 general practices, in all five northeast London boroughs. The secondary outcome was recorded new DVA cases in the electronic medical record in two boroughs. Data was analysed using an interrupted time series with a mixed effects Poisson regression model.

**Results:**

In the 144 general practices in the four implementation boroughs, there was a significant increase in referrals received by DVA workers—global incidence rate ratio of 30.24 (95% CI 20.55 to 44.77, *p* < 0.001). There was no increase in the 61 general practices in the other comparator borough (incidence rate ratio of 0.95, 95% CI 0.13 to 6.84, *p* = 0.959). New DVA cases recorded significantly increased with an incident rate ratio of 1.27 (95% CI 1.09 to 1.48, *p* < 0.002) in the implementation borough but not in the comparator borough (incidence rate ratio of 1.05, 95% CI 0.82 to 1.34, *p* = 0.699).

**Conclusions:**

Implementing integrated referral routes, training and system-level support, guided by a national health-focused DVA organisation, outside of a trial setting, was effective and sustainable at scale, over four years (2012 to 2017) increasing referrals to DVA workers and new DVA cases recorded in electronic medical records.

## Background

Domestic violence and abuse (DVA) is a global health and societal concern [1]. It is a violation of human rights which damages health, posing a challenge to public health and clinical practice [2, 3]. World Health Organization [4], National Institute for Health and Care Excellence guidelines [5] and the most recent UK Government Department of Health & Social Care guidance [6] all support greater health sector involvement, to improve the healthcare of those affected by DVA. Most recently, the Guttmacher-Lancet Commission placed detection and management of gender-based violence, central to delivering sexual and reproductive health for all [7]. In most settings, including high-income countries, healthcare is still not responding adequately to violence against women, which will most commonly be DVA [8]. This response’s contribution to delivering better healthcare for all has not been realised [9]. Ten years following the completion of our cluster randomised controlled trial demonstrating the effectiveness of IRIS—Identification and Referral to Improve Safety of women affected by DVA—a complex, system-level, training, support and referral programme, designed to improve the primary healthcare response to DVA [10], we now report an analysis of a population-wide implementation evaluation of IRIS, over four years (2012 to 2017).

A previous systematic review of interventions to improve clinicians’ response to DVA [11], updated and developed to focus only on high-quality, low risk of bias trials, measuring outcomes changing either clinicians’ behaviour (identifying and referring cases) or patient outcomes, found three studies [ 10, 12, 13], two of which [10, 12] were identified in the original systematic review [11]. These higher-quality studies supported the review’s overall conclusion that the best strategy to improve clinicians’ responses to DVA is training clinicians, face-to-face (as opposed to online interventions that have not been evaluated for actual behaviour change)—with optimum duration and precise elements of training undefined—whilst increasing all staff’s understanding of DVA (not just awareness). Two of these studies supported a clinical case-finding approach [10, 12], with one intervention including system-supportive interventions–incorporating clinical reminders on identifying DVA within consultations and direct access for women to DVA services within the safe space of healthcare settings [10]. The most recent third study did not support a system of care for postal screening, followed by general practice invites to women affected by DVA [13]. Out of the three trials, the IRIS trial intervention was unique as it nurtured and established greater health services’ participation by linking NHS statutory primary care to the multi-sectoral response to violence against women, via the DVA agency-employed advocate educator [10]. The IRIS trial, based on 24 intervention general practices with controls, in two English cities (London and Bristol), over one year, reported an increase in recorded DVA identification (three-fold), referrals made for advocacy (seven-fold) and referrals’ discussion (22-fold). The intervention consisted of training sessions for the whole practice team, a focus on clinically relevant DVA case finding, using the accurate Humiliation, Afraid, Rape, Kick (HARK) tool [14, 15], embedded in the electronic medical record (EMR), reminding clinicians to listen for and consider DVA. Qualitative analysis nested within the original IRIS trial showed that women had positive experiences about being asked about abuse, when clinically indicated by health professionals and contact with DVA advocates [16]. Health professionals viewed IRIS as an acceptable intervention but were concerned that their four hours of training was lengthy [17]. Modelling with trial data showed that the IRIS intervention was cost-effective [18].

Following the trial, the IRIS intervention has been funded in 41 English and Welsh, urban and rural sites. In one quarter of sites, funding has stopped. Secure longer-term funding allowing more stable healthcare services’ involvement, at a much larger scale than at present, requires more evidence that the intervention is effective outside the trial context. We hypothesised that this system-level programme would be effective and sustained outside of a trial.

## Methods

The aim of this study was to determine whether IRIS is effective when implemented in primary care outside a trial setting, in multiple sites, over four years. For full details of study design, participants, procedures, interventions, and data sources, see our protocol paper [19]. We have used the revised Standards for QUality Improvement Reporting Excellence (SQUIRE 2.0) [20] for reporting this evaluation (see Additional file 1).

### Study design and participants

Observational implementation study [21] conducted a segmented regression interrupted time series (ITS) analysis [22] of routinely collected longitudinal data, including over a baseline year prior to implementation, from general practices that implemented the IRIS programme in four boroughs and general practices invited to attend a DVA education session, more akin to *usual care* in a neighbouring borough.

All northeast London boroughs that funded IRIS were included in this study (A, B, C and D). The one neighbouring northeast London borough (E) that declined to fund IRIS instead organised lower cost DVA education sessions. Hence, E served as a contemporaneous non-random comparator borough. Randomisation was not possible. All data from the implementation boroughs and the comparator borough were collected within the same 53-month study period (from November 2012 to the end of March 2017). All general practice-registered women aged 16 and above were eligible for inclusion in this study.

This study used only anonymised observational data, obtained assessing routine clinical care so individual consent of women was not required. Prior to the start of this study, advice was sought from the research ethics committee chair with written approval given for this work as an evaluation of routine care.

### Processes

Our case definition of DVA against women was physical, sexual, psychological or emotional abuse, including threatening behaviour, either from a partner, ex-partner or an adult family member, currently or historically. In all areas, women affected by DVA could be identified by a clinician and offered a referral to a DVA worker. Women could also self-refer if they saw publicity material displayed within a practice.

The IRIS model has five core components: 1. practice-based training—two two-h clinical sessions, one h for administration staff and two-h refresher training, offered two years later; 2. a local GP appointed as an IRIS clinical lead delivers DVA training relevant to providing clinical care; 3. a prompt to consider DVA, with HARK, in the EMR triggered by codes for health conditions or symptoms associated with DVA; 4. a named DVA specialist, the IRIS advocate educator, employed by and based at a local DVA service jointly delivers training (as above) with the clinical lead; and 5. this IRIS advocate educator receives referrals directly from trained clinicians and sees patients affected by DVA, usually within the practice, unlike usual primary care settings, dispensing expert advocacy and ensuring direct access for women to specialist abuse services. We have described the IRIS model using the TIDieR checklist [23] (see Additional file 2). IRISi (IRIS interventions), a national, health-focused DVA organisation, brings experience and knowledge of starting up 37 other local IRIS services, facilitating negotiations and strengthening the National Health Services’ (NHS) relationship with DVA agencies. IRISi using data (nationally collected, analysed and monitored) steers implementation, on-going support, staff recruitment and training, for each local IRIS service. Staff mix is crucial as reported in the implementation process evaluation [24].

The other DVA education model delivered in the comparator borough comprised a session, conveying information about DVA, located in community venues (not in general practices), led by a local DVA service provider (not delivered by clinicians), no prompt to consider DVA within the EMR and no named advocates receiving referrals. This is the most usual type of DVA education for healthcare providers in England and Wales and represents *usual care*. Globally, support to provide a proficient healthcare response to DVA is even more rudimentary or absent.

### Outcomes

Our primary outcome was the daily number of referrals received by DVA workers per 1000 women registered in a general practice. This is an intermediate outcome measure, on a causal pathway towards decreased incidence of DVA, possibly better mental health and improved quality of life [25]. In boroughs A and D, there was one sole DVA service provider, receiving all referrals, from which data were collected. In boroughs B and C, there were several DVA service providers; in these, we collected referral data from the major DVA agency, receiving more than 80% of referrals in that area. Smaller DVA service providers, receiving less than 20% referrals, in these two areas only, did not routinely record their client’s registered general practice. In the comparator borough (E), there was a ‘One-Stop Shop’ through which all referrals to multiple DVA service providers were received, and from which we collected referral data.

Our secondary outcome measure was the number of women in whose EMR first usage of a DVA identification code occurred, per 1000 women registered in a general practice, corresponding to the incidence of DVA cases identified and recorded by general practices. These data were only available in boroughs C and E, as they had data sharing agreements. The type of contact that referred women had with IRIS advocate educators was also collected.

### Statistical analysis

#### Power calculation

Following the introduction of IRIS, we wanted to be able to detect a doubling of the referral rate, thus an incident rate ratio (IRR) of at least two, with 90% power, at the 5% significance level. IRRs compare the periods before and after the first DVA session delivery. They are based on regression coefficients. The regression coefficient is the constant that represents the rate of change of referrals received by DVA workers from clinicians, as a function of change over time in days, before and after implementation of the DVA package. For the power calculation, we assumed that the implementation of IRIS by each general practice occurred at different times uniformly over the time period. We anticipated that there would be approximately 180 general practices in the ITS evaluation. Based on the data from the original IRIS trial [10], we estimated that with an average of 3000 eligible women per practice, a typical practice average referral rate at baseline of 4.5 referrals made and received by the DVA agency per 100,000 registered women aged 16 years and above, per month, with this rate varying between practices with a 95% normal range of 1.7 to 12.2 per 100,000 per month. Simulations of monthly counts with a Poisson distribution showed that 17 months of data were required to detect a doubling of the referral rate. We collected data over at least 24 months, in order to ensure that any seasonality in referral rates did not impact on our results. Simulation using the SimSam package in Stata was used to calculate the sample size [26]. For this sample size calculation and the code that enables it to be reproduced, see Additional file 3.

For the analysis, the effect of both DVA programmes was defined by the delivery of the first training session for IRIS and the information session for the other DVA education model. Hence, our impact model assumed a step change model, with these educational sessions if effective thought to be key to a prompt outcome response, with an immediate and on-going impact. This was examined using practice-level daily data, before and after the date of these sessions respectively, to detect whether either DVA programme had an effect significantly greater than the underlying secular trend. The primary and secondary outcome measures were analysed using an ITS segmented approach with a mixed effects Poisson regression model. This ITS model included a random effect of practice and fixed effects of education (pre or post in any given day in any given practice), the gradient of the underlying time trend, the change in gradient following the teaching, site (borough), and month and day of the week to allow for any seasonal effect of time. In order to model over-dispersion of daily frequencies, we included a random effect of day nested within the random effect of practice in our Poisson regression model. To control for the available population size at any one time, we adjusted for the log-transformed number of women aged 16 years and above registered at a practice for that quarter, as an offset variable in the analysis. The general practices that did not receive any DVA session, had an unknown number of women registered, had missing training dates or had closed were excluded from the statistical analysis; as were the observations on the day of DVA session delivery, as uncertain whether these observations occurred before or after the education sessions.

A global estimate of the IRR was calculated by fitting an ITS regression to the combined data from all four boroughs where IRIS was implemented. A *z* test was used to test whether the coefficient from the ITS Poisson regression model was different from 0 (or whether the ITS Poisson regression model’s exponentiated coefficient was different from one), generating a *p* value. ITS regression models were also fitted separately for each of the five boroughs. The presence of autocorrelation was assessed through sensitivity analysis using clustered standard errors in the ITS regression model. General practices were defined as the cluster.

#### Sub-group analyses

To determine whether there was heterogeneity in the treatment effect (i.e. impact of IRIS implementation) across the different boroughs, the effect estimates were graphically displayed in a forest plot, and Cochran’s test of heterogeneity [27] was calculated. The same approach was taken to explore whether the IRIS implementation effect differed dependent upon a practice’s level of deprivation, using the Index of Multiple Deprivation, for which “0” denotes the least deprivation and “100” the most deprivation [28]. For the purpose of this analysis, deprivation score was grouped into three (scores 10 to ≤ 30, > 30 to < 30.65, > 30.65), with the category boundaries chosen to create an equal number of practices in each group. The effect estimates were also graphically displayed in a forest plot, and Cochran’s test of heterogeneity [27] was calculated. All sub-group analyses were pre-specified and done in Stata V14 (StataCorp LP, College Station, TX).

## Results

Table 1 shows largely comparable characteristics, including population size, of the participating five boroughs (A, B, C, D and E). The general practices that were included in the ITS analysis were consistently less deprived than all general practices in each of the five boroughs. All the boroughs have diverse populations with substantial proportions of non-white ethnicity, with the highest proportion in the comparison borough.

**Table 1:** Characteristics of five participating boroughs (A, B, C, D and E)

London borough	Sub-region	Population characteristics				Recorded crime and incidents	
		Female, thousands	Mean Index of Multiple Deprivation of all general practices	Mean Index of Multiple Deprivation of only general practices included in the analysis	Non-white ethnicity, %	Domestic incidents per 1000 female population	Domestic homicides 2009–2014, *n*
A	North	129.8	48	30	39.0	53.5	6
B	North	96.9	33	29	33.7	42.6	2
C	East	105.2	36	31	54.8	75.7	10
D	North	91.6	34	23	31.8	60.3	4
E	East	116.8	48	27	71.0	71.1	11

All data relates to financial year 2013/2014 with data source Mayor of London Office for Policing and Crime—except Index of Multiple Deprivation (IMD) is the 2015 score [28]

Table 2 shows a summary of the data collected, with absolute numbers of referrals received by DVA service providers from general practices, new DVA cases identified and recorded by general practices and explanation of missing data. In the boroughs where IRIS was implemented (A, B, C and D), 144 general practices were funded to receive IRIS training. Twenty-two general practices did not receive it. Three practices had an unknown number of women registered. Two practices had missing training dates. One practice closed. Hence, 116 practices (81%) were included in the analysis. In borough E, 27 general practices out of 61 sent no staff to their DVA session, so 34 (56%) practices were included in the analysis. The IRIS programme was implemented in northeast London from November 2013. IRIS continued to be funded in all four implementation boroughs (A, B, C and D) at the end of the data collection on 31 March 2017. In the comparator borough E, the DVA information sessions were delivered from May 2014. The data collection period included the year preceding the implementation, in all five boroughs. This was a total of 53 months, from November 2012 to the end of March 2017.

**Table 2:** Data summary—referrals received by DVA service providers from general practices and new DVA cases’ recorded identification by general practices

Boroughs	A	B	C	D	E
Number of practices for which DVA service funded	33 (50%)	39 (100%)	37 (100%)	35 (100%)	61 (100%)
Number of practices excluded (reasons)	2 (6%) (both trained—1 closed, 1 missing list size data)	9 (23%) (6 untrained; 3 trained—2 missing training dates, 1 very irregular list size data)	5 (13%) (5 untrained)	12 (34%) (11 untrained; 1 trained—very irregular list size data)	27 (44%) (27 untrained)
Number of practices included	31 (94%)	30 (77%)	32 (87%)	23 (66%)	34 (56%)
Observation period (number of days)	15 November 2012 to 31 March 2017 (1598)	14 March 2013 to 31 March 2017 (1479)	02 October 2013 to 31 March 2017 (1277)	29 January 2014 to 31 March 2017 (1158)	01 January 2014 to 31 December 2015 (730*)
First session delivery date	15 November 2013	14 March 2014	02 October 2014	29 January 2015	15 May 2014
Number of referrals received by DVA service providers from general practices	278	270	394	123	41
Number of referrals excluded (reason)	13 (5%) (1 on same day as training; 12 from untrained practices or from two excluded practices—as above)	7 (3%) (3 on same day as training; 1 from an excluded practice; 3 from practices outside of Camden)	0 (0%)	3 (2%) (2 from untrained practices; 1 from an unknown practice)	22 (54%) (20 from untrained practice; 2 from unknown practices)
Mean no. of referrals per day (variance)	0.005 (0.006)	0.006 (0.006)	0.01 (0.01)	0.005 (0.005)	0.0008 (0.0008)
Number of new DVA cases recorded identification by general practices	–	–	2808	–	2260
Number of DVA identification codes excluded (reason)		–	265 (2 on same day as training; 19 no accompanying date provided; 244 from excluded practices)	–	913 (4 no accompanying date provided; 909 from excluded practices)
Mean no. of new DVA cases identified per day (variance)	–	–	0.064 (0.071)	–	0.03 (0.03)

*The observation period in borough E for referrals received is smaller than that for DVA codes (for which it was 1417 days, from 15 May 2013 to 31 March 2017)

**Fig. 1:**
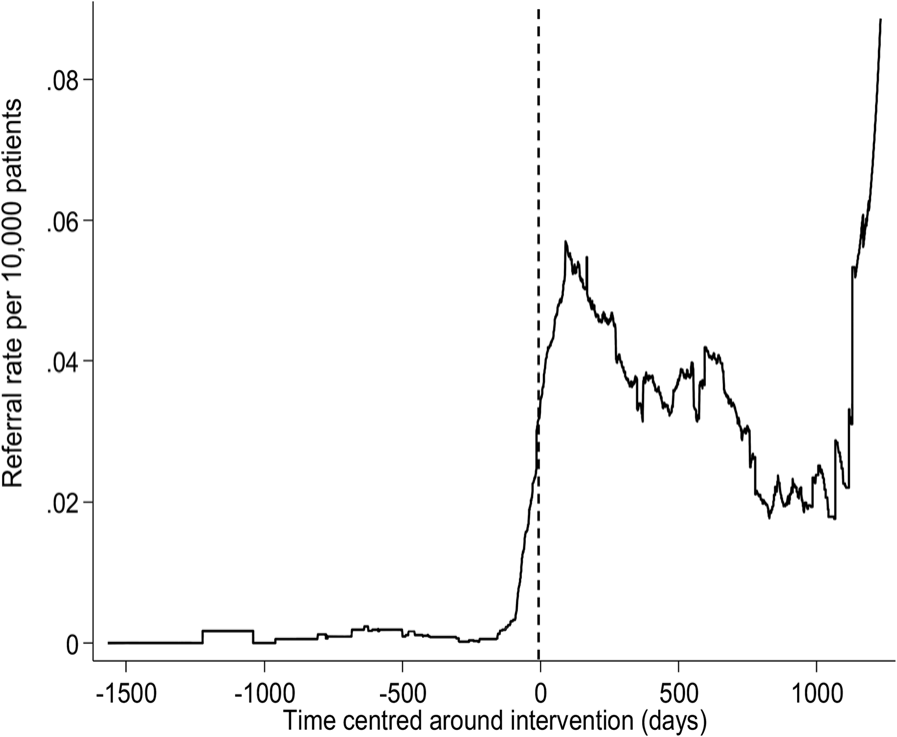
Average daily referral rate per 1000 women registered, in A, B, C and D, with time centred around the point of their first IRIS session delivery

**Fig. 2:**
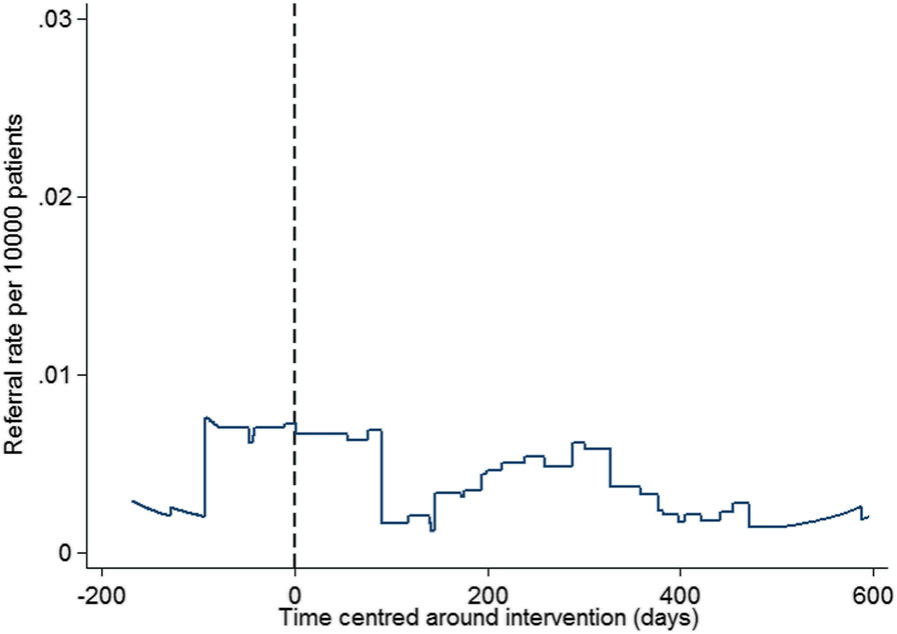
Average daily referral rate per 1000 women registered in E, with time centred around the point of their DVA session delivery

**Fig. 3:**
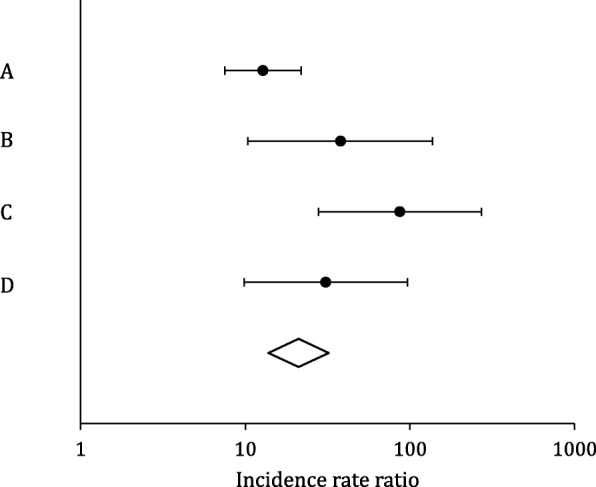
Forest plot comparing the estimated effect of IRIS first training session delivery on referrals received, in A, B, C and D

**Fig. 4:**
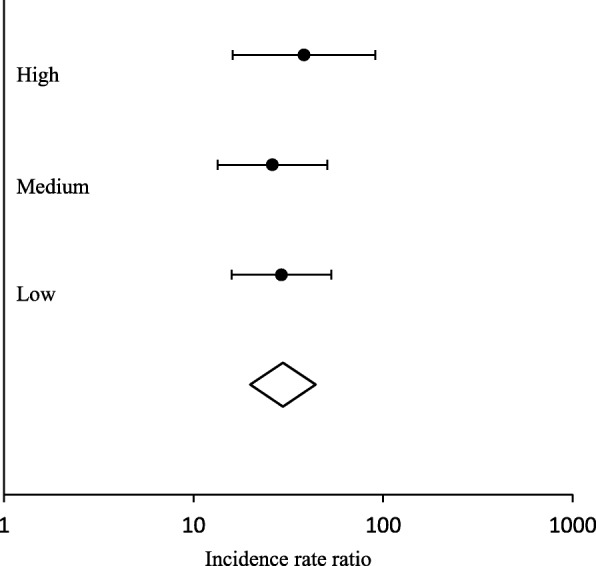
Forest plot comparing the estimated effect of IRIS first training session delivery on referrals received, across three deprivation groups in A, B, C and D. Using the Index of Multiple Deprivation, for which “0” denotes the least deprivation and “100” the most deprivation [28]. For the purpose of this analysis, category boundaries were chosen to create an equal number of practices in each group, with deprivation score grouped into three scores: 10 to ≤ 30, low deprivation score; > 30 to < 30.65, medium deprivation score; > 30.65, high deprivation score)

The global IRR for referrals received in the four boroughs where IRIS was implemented was 30.24 (95% CI 20.55 to 44.77, *p* < 0.001). Table 3 shows the IRRs for referrals received in all five boroughs with the delivery of their separate DVA sessions. The IRRs for the individual boroughs (A, B, C and D) are consistent with the global IRR, representing a large increase in the number of referrals received with the first training session delivery, in contrast to borough E that has no improvement in referrals received with its session delivery (IRR 0.95, 95% CI 0.13 to 6.84, *p* = 0.959). Figure 1 shows the average daily referral rate per 1000 women registered in the IRIS boroughs’ general practices (A, B, C and D), plotted against time centred around the day of delivery of their first IRIS training session, illustrating the large effect of IRIS implementation on referrals, and post-implementation shows that IRIS has a sustained effect on the increased numbers of referrals received. This scatter diagram is consistent with our chosen step change impact model, supporting that this model fitted the data well across all four boroughs. Figure 2 shows that the average daily referral rate per 1000 women registered in borough E, before and after the day of delivery of their DVA session, did not significantly differ. The result from the sensitivity analysis with clustered standard errors was IRR 28.78 (95% CI 16.38 to 50.56, *p* < 0.001). These results allow for potential autocorrelation and are consistent with our main findings. The estimated effect of IRIS’ first training session delivery on referrals received is not significantly different in the four implementation boroughs (see Fig. 3), nor affected by socioeconomic status, as reflected by the deprivation level of each practice (see Fig. 4): low deprivation (score 10 to ≤ 30, medium (score > 30 to < 30.65) and high (score > 30.65). Cochran’s heterogeneity test shows there is no significant heterogeneity between boroughs or deprivation score level, producing *p* values of 0.460 and 0.850 respectively. IRISi reports that nationally, IRIS advocate educators have contact with and support 85% of the patients that are referred to them from general practice (IRIS National Report 2018).

**Table 3:** Referrals received by DVA service providers from general practices—incident rate ratios (IRR) with sessions’ delivery

	A (*n* = 31)		B (*n* = 30)		C (*n* = 32)		D (*n* = 23)		E (*n* = 34)	
	IRR (95% CI)	*p*	IRR (95% CI)	*p*	IRR (95% CI)	*p*	IRR (95% CI)	*p*	IRR (95%)	*p*
Underlying time trend (IRR per day)	1.00 (0.99, 1.00)	0.157	1.00 (0.99, 1.01)	0.100	0.99 (0.99, 1.00)	0.524	0.99 (0.99, 1.00)	0.921	1.00 (0.98, 1.02)	0.681
Average effect of session delivery	12.82 (7.51, 21.88)	< 0.001	37.92 (10.36, 138.82)	< 0.001	86.93 (27.81, 271.73)	< 0.001	30.83 (9.82, 96.76)	< 0.001	0.95 (0.13, 6.84)	0.959
Change in gradient following session delivery	0.99 (0.998, 0.999)	0.026	0.99 (0.98, 0.99)	0.99	1.00 (0.99, 1.00)	0.853	0.99 (0.99, 1.00)	0.663	0.99 (0.98, 1.02)	0.675

Table 4 shows IRRs for new DVA cases identified and recorded by general practices in boroughs C and E with the delivery of DVA sessions. The IRR in borough C was 1.27 (95% CI 1.09 to 1.48, *p* < 0.002), demonstrating that there is a significant increase in the number of new DVA cases identified and recorded by general practices, on average with the first IRIS training session delivery. This increase is not seen with the other DVA model’s information session delivery in borough E (IRR = 1.05, 95% CI 0.82 to 1.34, *p* = 0.699).

**Table 4:** New DVA cases identified and recorded by general practices—incident rate ratios (IRR) with sessions’ delivery

	C (*n* = 32)		E (*n* = 34)	
	IRR (95% CI)	*p*	IRR (95% CI)	*p*
Underlying time trend (IRR per day)	0.99 (0.99, 1.00)	0.322	0.99 (0.99, 1.00)	0.118
Average effect of session delivery	1.27 (1.09, 1.48)	0.002	1.05 (0.82, 1.34)	0.699
Change in gradient following session delivery	0.99 (0.99, 1.00)	0.193	0.99 (0.99, 1.00)	0.176

## Discussion

Our interrupted time series analysis of data from five London boroughs shows sustained effectiveness of IRIS implementation over four years, with clinicians significantly increasing referrals to DVA service providers. A different DVA education model, focussed on conveying information, in a comparable neighbouring borough, more representative of usual care in the UK, did not increase referrals. The difference between the boroughs where IRIS was implemented and the other comparator borough was substantial, with the global IRR in the former being 30.24 whilst the IRR in the latter 0.95.

In the boroughs where IRIS was implemented, the preceding time trend confirms that this large step-wise increase in referrals with IRIS training delivery was not due to an underlying temporal trend. The other borough’s time trend before and after the delivery of its DVA session showed no equivalent step-wise change. Following the step-wise increase in the boroughs where IRIS was implemented, the higher referral rate continued, with no significant differences across the four boroughs, and did not vary by deprivation status of the practice populations.

This study was sufficiently powered to detect a clinically important increase in referrals. In the four boroughs where IRIS was implemented, with the first IRIS training session delivery, each IRR increases (far excess of doubling), reflecting the large increase in referrals made by individual general practices with IRIS implementation—a large relative difference in referral numbers. The wide confidence intervals of these IRRs, in these four boroughs, reflects the uncertainty about the exact point estimate—but examining the lower limits of all four of these confidence intervals (lowest 7.51 in borough A), there is always much more than a doubling in the referrals received by the DVA agencies from general practices. The relative increase in referrals received with IRIS implementation was much larger than the relative increase in recorded identification of new DVA cases. The IRIS advocate educators’ on-going support, consultancy and interactions with the whole practice team, including refresher training, conceivably have a greater impact on referrals than on clinicians’ recording of new DVA cases. The absolute numbers of referrals made with IRIS implementation are much smaller than the numbers of new DVA cases recorded within the EMR (in borough C 394 referrals vs 2808 cases over 3½ years), as the latter is dependent on many external factors, including communication with other statutory organisations such as the police, with a child safeguarding focus. In turn, the numbers of new DVA cases recorded are smaller than the estimates of DVA prevalence in community or healthcare settings—using a one-year prevalence estimate of 4.2% [29], in borough C, there would be ~ 4418 cases. This difference is unsurprising, as most DVA is not shared outside of the home. This also indicates potential gains from continuing to improve the healthcare response to DVA.

Study strengths include no exclusion of potential borough sites, outcomes measured on a daily basis and sustainability examined robustly. Data collection in the four boroughs where IRIS was implemented surpassed the minimum of 24 months pre-specified in the sample size calculation. Collecting more data than was required, over a longer time period, gave us the capability to evaluate the sustainability of an effect over time, rather than only measuring the initial change in referral rate following delivery of IRIS training. Data accuracy was validated by coinciding sources if possible (for example, national data compared to local data). The primary outcome measure, referrals received by DVA service providers from general practices, was potentially susceptible to ascertainment bias, since the agencies knew they were part of the IRIS programme. Data collection was likely to be the most complete in the comparison borough, as all referrals were collected at a single site, the One-Stop Shop, ensuring ascertainment bias was less likely here than the IRIS implementation boroughs. Therefore, the comparison of effect differences was conservative. Referrals actually received by DVA service providers, as opposed to those reportedly made by clinicians, have proved to be the most reliable measure of a healthcare system responding to DVA [10]. This observational study’s community setting, in routine general practice, with borough-wide implementation, with the IRIS effect not significantly different in the four implementation boroughs with diverse populations, makes its results generalisable to similar settings in the UK and potentially to comparable primary care systems in high-income countries. For lower-income countries [30], this healthcare response to DVA is modifiable, if the health setting is safe, using existing community resources if DVA service infrastructure is absent.

Study limitations include not measuring direct patient-level outcomes, using exposure to DVA advocacy as a proxy measure, outcome assessors not blinded to data origin and no double entry of data. DVA service providers generously shared their data—but we did not have complete access to the entirety of all their data sets so we were unable to assess its completeness. In two of the IRIS implementation boroughs, smaller DVA service providers, receiving less than 20% referrals, did not routinely record their client’s registered general practice, precluding their data from the analysis.

Our study did not have any randomisation component. However, overall, the causal inference that IRIS causes clinicians’ behaviour to change, increasing the referrals received by DVA service providers, is strengthened, as it is now supported by both the preceding randomised trial, as well as this ITS. Furthermore, the ITS analysis of the comparator borough E without IRIS, showing no increase in the referral rate, is also evidence that it was IRIS that was responsible for the increase in referrals received of women affected by DVA. Unlike other DVA work, this study is linked to a cost-effectiveness analysis, estimating that IRIS outside the trial setting is cost-effective from a health service and societal perspective, moreover good value for the NHS and cost saving for society (the incremental net monetary benefit was £22 and £42 respectively) [31].

## Conclusion

Our study provides evidence that a system-level programme that embeds direct referral pathways to specialist DVA agencies within health services, underpinned by training of clinicians and their teams, including on-going reinforcement strategies and processes in place, from the outset, improves the healthcare response to DVA. This can be successfully implemented, with continued effectiveness and sustained over four years simultaneously in multiple areas, outside of a trial setting. Global health policy is responding to the challenge of ensuring that health services understand their role in providing effective and compassionate healthcare in the context of DVA [4]. For health professionals to engage effectively, internationally as well as in the UK, further resources are required (within health and for specialist DVA agencies), best care reconsidered and new grass root adopters of DVA programmes respected [32]. As a healthcare response to DVA is implemented, the next step is translation research questions, directly assessing population health impact [33]. This study provides further evidence for funding of healthcare-based programmes that combine direct referral pathway to specialist DVA services with training and on-going reinforcement.

## Supplementary information

**Additional file 1.** Revised Standards for QUality Improvement Reporting Excellence (SQUIRE 2.0) – checklist for evaluation reporting.

**Additional file 2.** Template for intervention description and replication (TIDieR) – checklist for IRIS description.

**Additional file 3.** Sample size calculation with code enabling it to be reproduced.

## Data Availability

The datasets generated, used and analysed during this study are available from the corresponding author on reasonable request. The datasets are contained in the Barts Cancer Centre repository. A short video about this implementation research’s findings can be found here: https://drive.google.com/open?id=1Wc9hXoF06qDhFD2ujgQJBDgJe06nc0jc. All the people in the video have consented to their inclusion and the video being made public.

## Abbreviations

CEG: Clinical Effectiveness Group
DVA: Domestic violence and abuse
EMIS: Egton Medical Information Systems
EMR: Electronic medical record
GP: General practitioner
HARK: Humiliation, Afraid, Rape, Kick tool
IRIS: Identification and Referral to Improve Safety
IRISi: Identification and Referral to Improve Safety interventions—this is a social enterprise established to promote and improve the healthcare response to gender-based violence (for further information, see www.irisi.org)
IRR: Incident rate ratio
ITS: Interrupted time series
NHS: National Health Service
NICE: National Institute for Health and Care Excellence
UK: United Kingdom
WHO: World Health Organization
