# Supplementary material for: Improving the healthcare response to domestic violence and abuse in UK primary care: interrupted time series evaluation of a system-level training and support programme
Source: BMC Med. 2020 Mar 5;18:48. doi: 10.1186/s12916-020-1506-3 (PMC7057596; doi:10.1186/s12916-020-1506-3)
Supplement: Supplementary file 1 — Additional file 1. Revised Standards for QUality Improvement Reporting Excellence (SQUIRE 2.0) – checklist for evaluation reporting. [file 12916_2020_1506_MOESM1_ESM.docx]

**IRIS description**

| **N** | **Item** | **Description** Information contained under Processes, pages five to six.  Also further details contained in published Protocol paper (under Population/participants & Intervention; & Appendix Additional files – IRIS publicity materials). |
| --- | --- | --- |
|  | **BRIEF NAME** |  |
| **1.** | Provide the name or a phrase that describes the intervention. | IRIS - Identification and Referral to Improve Safety of women affected by domestic violence & abuse |
|  | **WHY** |  |
| **2.** | Describe any rationale, theory, or goal of the elements essential to the intervention. | The theoretical framework of the training is based on educational outreach, adult learning theory and peer influence. It was developed using the MRC framework for complex interventions. |
|  | **WHAT** |  |
| **3.** | Materials: Describe any physical or informational materials used in the intervention, including those provided to participants or used in intervention delivery or in training of intervention providers. Provide information on where the materials can be accessed (e.g. online appendix, URL). | Training pack.  Flowcharts showing simple referral pathways.  Presentations.  HARK template. An electronic prompt, that is incorporated into the electronic medical record, triggered by codes for health conditions or symptoms associated with DVA, such as insomnia, anxiety/depression, pelvic pain and unexplained pain, reminds clinicians to ask about DVA; with the HARK (Humiliation, Anger, Rape, Kick) template, for recording presence of specific DVA dimensions (encouraging clinicians to (i) assess the immediate safety of the woman and any children, (ii) offer referral, (iii) review within general practice). Would need to contact IRISi (via [www.irisi.org](http://www.irisi.org)) for access to these materials.  Patient materials – IRIS cards and posters to display in practices (can be accessed via the published Protocol paper, in Appendix Additional files). |
| **4.** | Procedures: Describe each of the procedures, activities, and/or processes used in the intervention, including any enabling or support activities. | Training: slide presentation, questions & answers, role play.  On-going support, refresher training and consultancy: for the entire practice team, on a day-to-day basis when in the practice (preferably attending regular quarterly practice meetings), by phone and email. |
|  | **WHO PROVIDED** |  |
| **5.** | For each category of intervention provider (e.g. psychologist, nursing assistant), describe their expertise, background and any specific training given. | 1. A named DVA specialist – an IRIS advocate educator - employed and based in a local DVA service (known as the Host Agency), delivers training and also receives referrals from clinicians, responsible for a caseload of work, providing on-going support, refresher training and consultancy for the entire practice team. From a DVA advocacy background. 2. A local GP, interested in DVA, appointed as an IRIS clinical lead delivers clinical training alongside an IRIS advocate educator.   Both would receive the IRIS Train the Trainers training. |
|  | **HOW** |  |
| **6.** | Describe the modes of delivery (e.g. face-to-face or by some other mechanism, such as internet or telephone) of the intervention and whether it was provided individually or in a group. | General practice based DVA training, with two two-hour clinical sessions, one hour for administration staff and two-hour refresher training for the whole practice team. |
|  | **WHERE** |  |
| **7.** | Describe the type(s) of location(s) where the intervention occurred, including any necessary infrastructure or relevant features. | Training in general practice.  On-going support either in general practice or remotely.  DVA advocacy at a location safe and convenient to patient (e.g. in general practice, host agency or other location) |
|  | **WHEN and HOW MUCH** |  |
| **8.** | Describe the number of times the intervention was delivered and over what period of time including the number of sessions, their schedule, and their duration, intensity or dose. | Two two-hour clinical sessions  One one-hour session for non-clinical staff.  Additionally communication about individual referrals – when the referral is made and also ongoing feedback about the referral between individual clinicians & the advocate educator.  Two-hour refresher training beyond one year. |
|  | **TAILORING** |  |
| **9.** | If the intervention was planned to be personalised, titrated or adapted, then describe what, why, when, and how. | In each local area, a local IRIS steering group is formed that is responsible for the development and implementation of IRIS locally – for example, the tendering process that results in appointing a Host Agency who employs the advocate-educator. Hence the local IRIS intervention is adapted appropriately for that local area. |
|  | **MODIFICATIONS** |  |
| **10.^ǂ^** | If the intervention was modified during the course of the study, describe the changes (what, why, when, and how). | All local areas were encouraged by IRISi to stick to the five core components of IRIS (see pages 8 to 9). A five-item checklist, assessing the fidelity of IRIS, identified IRIS’ local adaptations – as described in the published protocol paper. |
|  | **HOW WELL** |  |
| **11.** | Planned: If intervention adherence or fidelity was assessed, describe how and by whom, and if any strategies were used to maintain or improve fidelity, describe them. | IRIS intervention fidelity was assessed by the five-item checklist - with plan to write up in a future paper. IRISi fostered best practice at each local site, encouraging that the five core components of the IRIS model are adhered to at all stages. |
| **12.^ǂ^** | Actual: If intervention adherence or fidelity was assessed, describe the extent to which the intervention was delivered as planned. | IRIS intervention fidelity was assessed by the five-item checklist - with plan to write up in a future paper. |

** **Authors** - use N/A if an item is not applicable for the intervention being described. **Reviewers** – use ‘?’ if information about the element is not reported/not sufficiently reported.

† If the information is not provided in the primary paper, give details of where this information is available. This may include locations such as a published protocol or other published papers (provide citation details) or a website (provide the URL).

ǂ If completing the TIDieR checklist for a protocol, these items are not relevant to the protocol and cannot be described until the study is complete.

* We strongly recommend using this checklist in conjunction with the TIDieR guide (see *BMJ* 2014;348:g1687) which contains an explanation and elaboration for each item.

* The focus of TIDieR is on reporting details of the intervention elements (and where relevant, comparison elements) of a study. Other elements and methodological features of studies are covered by other reporting statements and checklists and have not been duplicated as part of the TIDieR checklist. When a **randomised trial** is being reported, the TIDieR checklist should be used in conjunction with the CONSORT statement (see [www.consort-statement.org](http://www.consort-statement.org)) as an extension of **Item 5 of the CONSORT 2010 Statement.** When a **clinical trial** **protocol** is being reported, the TIDieR checklist should be used in conjunction with the SPIRIT statement as an extension of **Item 11 of the SPIRIT 2013 Statement** (see [www.spirit-statement.org](http://www.spirit-statement.org)). For alternate study designs, TIDieR can be used in conjunction with the appropriate checklist for that study design (see [www.equator-network.org](http://www.equator-network.org)).
