# Supplementary material for: Improving the healthcare response to domestic violence and abuse in UK primary care: interrupted time series evaluation of a system-level training and support programme
Source: BMC Med. 2020 Mar 5;18:48. doi: 10.1186/s12916-020-1506-3 (PMC7057596; doi:10.1186/s12916-020-1506-3)
Supplement: Supplementary file 2 — Additional file 2. Template for intervention description and replication (TIDieR) – checklist for IRIS description. [file 12916_2020_1506_MOESM2_ESM.docx]

| **Research and reporting methodology** |  | |  |
| --- | --- | --- | --- |
| Revised **Standards for QUality Improvement Reporting Excellence** (**SQUIRE 2.0**) publication guidelines |  | |  |
|  |  | |  |
| **Notes to authors** |  | |  |
| ▸ The SQUIRE guidelines provide a framework for reporting new knowledge about how to improve healthcare. |  | |  |
| ▸ The SQUIRE guidelines are intended for reports that describe system level work to improve the quality, safety and value of healthcare, and used methods to establish that observed outcomes were due to the intervention(s). |  | |  |
| ▸ A range of approaches exists for improving healthcare. SQUIRE may be adapted for reporting any of these. |  | |  |
| ▸ Authors should consider every SQUIRE item, but it may be inappropriate or unnecessary to include every SQUIRE element in a particular manuscript. |  | |  |
| ▸ The SQUIRE glossary contains definitions of many of the key words in SQUIRE. |  | |  |
| ▸ The explanation and elaboration document provides specific examples of well-written SQUIRE items and an in-depth explanation of each item. |  | |  |
| ▸ Please cite SQUIRE when it is used to write a manuscript. |  | |  |
|  |  | |  |
| **Text section and item name** | | **Page/line no(s).** | |
|  | | **info is located** | |
| **Title and abstract** | |  | |
| 1. **Title** | | Page one | |
| Indicate that the manuscript concerns an initiative to improve healthcare (broadly defined to include the quality, safety, effectiveness, patient-centredness, timeliness, cost, efficiency and equity of healthcare). | | Title starts with the word “Improving…” | |
|  | |  | |
| 2. **Abstract** | | Page two | |
| a. Provide adequate information to aid in searching and indexing. | | Yes | |
| b. Summarise all key information from various sections of the text using the abstract format of the intended publication or a structured summary such as: background, local problem, methods, interventions, results, conclusions. | | Yes – structured summary sub-sections used as requested. | |
|  | |  | |
| **Introduction: Why did you start?** | | Page three | |
| 3. **Problem description** - Nature and significance of the local problem. | | First paragraph | |
| 4. **Available knowledge** - Summary of what is currently known about the problem, including relevant previous studies. | | Second paragraph | |
| 5. **Rationale** - Informal or formal frameworks, models, concepts and/or theories used to explain the problem, any reasons or assumptions that were used to develop the intervention(s) and reasons why the intervention(s) was expected to work | | Third paragraph of Intro Background (on page four): Rationale – to secure longer term funding | |
| 6. **Specific aims** - Purpose of the project and of this report. | | Page four – last sentence of Background on hypothesis; with first sentence of Method listing study aim. | |
|  | |  | |
| **Methods: What did you do?** | | Pages four to nine | |
| 7. **Context** - Contextual elements considered important at the outset of introducing the intervention(s). | | Page five - all boroughs in urban northeast London; none excluded. | |

| 8. **Intervention(s)** | Page five to six: described under “Processes” |
| --- | --- |
| a. Description of the intervention(s) in sufficient detail that others could reproduce it. | Page five - the five core components of the IRIS model, with the role of the national health focused DVA organisation described in detail. IRIS model has also been reported using the TIDieR checklist. Further detail with IRIS materials are published in Protocol paper. Reproducing training slides & IRISi role would require communicating with IRISi via website (details on page fifteen). |
| b. Specifics of the team involved in the work. | Yes (page six) |
| 9. **Study of the intervention(s)** |  |
| a. Approach chosen for assessing the impact of the intervention(s). | Page five: Study design |
| b. Approach used to establish whether the observed outcomes were due to the intervention(s). | Multiple aspects of the Methods establish that observed outcomes were due to IRIS – these approaches are summarised in Discussion, page eleven, second paragraph and page 13, last paragraph. |
| 10. **Measures** |  |
| a. Measures chosen for studying processes and outcomes of the intervention(s), including rationale for choosing them, their operational definitions and their validity and reliability. | Page six to seven: Outcomes – includes rationale for using them; data accuracy validity and reliability covered under Discussion. Study strengths (page twelve to thirteen). |
| b. Description of the approach to the ongoing assessment of contextual elements that contributed to the success, failure, efficiency and cost. | N/A – online access to medical records maybe an ongoing contextual element; not discussed due to focused nature of this paper. |
| c. Methods employed for assessing completeness and accuracy of data. | Data accuracy covered in Discussion Study strengths (page twelve to thirteen); with completeness in Study limitations (page thirteen) |
| 11. **Analysis** | Page seven to nine. |
| a. Qualitative and quantitative methods used to draw inferences from the data. | Yes – only quantitative methods presented in this paper. |
| b. Methods for understanding variation within the data, including the effects of time as a variable. | Yes – described under Stats Analysis (page eight, second paragraph) |
| 12. **Ethical considerations** - Ethical aspects of implementing and studying the intervention(s) and how they were addressed, including, but not limited to, formal ethics review and potential conflict(s) of interest. | Page five, third paragraph; and on page fifteen, under “Declarations.” |
|  |  |
| **Results: What did you find?** | Page nine to eleven |
| 13. **Results** |  |
| a. Initial steps of the intervention(s) and their evolution over time (eg, time-line diagram, flow chart or table), including modifications made to the intervention during the project. | Page nine, second paragraph describes data collected; & presented in Table 2 |
| b. Details of the process measures and outcomes. | Process measures covered in separate paper published (on Process Evaluation). Outcomes’ Results on page ten, Tables 3 & 4 and Figures 1 to 4 |
| c. Contextual elements that interacted with the intervention(s). | Table 1 describes context - “Characteristics of five participating boroughs”  (N/A – online access to medical records maybe a contextual element that affected outcome measures; not discussed due to focused nature of this paper & of less relevance to a global readership). |
| d. Observed associations between outcomes, interventions and relevant contextual elements. | Page ten – “*…a large increase in the number of referrals received with first training session delivery,…”* |
| e. Unintended consequences such as unexpected benefits, problems, failures or costs associated with the intervention(s). | Not discussed as n/a. |
| f. Details about missing data. | Described on page nine, in second paragraph of “Results” (& Table 2) |
|  |  |
| **Discussion: What does it mean?** | Page 11 to 14 |
| 14. **Summary** | Page 11, first paragraph |
| a. Key findings, including relevance to the rationale and specific aims. | Page 11, first paragraph |
| b. Particular strengths of the project. | Page 12 to 13, in fourth paragraph of Discussion, the study strengths are listed. |
|  |  |
| 15. **Interpretation** |  |
| a. Nature of the association between the intervention(s) and the outcomes. | Page 13 – last paragraph |
| b. Comparison of results with findings from other publications. | Page 13, last sentence of last paragraph, starting with “Unlike other DVA work,…” |
| c. Impact of the project on people and systems. | Page 14 – last paragraph. |
| d. Reasons for any differences between observed and anticipated outcomes, including the influence of context. | Not discussed. |
| e. Costs and strategic trade-offs, including opportunity costs. | Page 13, last sentence, discusses cost-effectiveness. |
|  |  |
| 16. **Limitations** |  |
| a. Limits to the generalisability of the work. | Page 13, last sentence of top paragraph covers generalisability of this work, including its limits. |
| b. Factors that might have limited internal validity such as confounding, bias or imprecision in the design, methods, measurement or analysis. | Page 13, paragraph on “Study limitations…” |
| c. Efforts made to minimise and adjust for limitations. | Page 12, last paragraph touches upon minimising data inaccuracy by validating using coinciding data sources. |
|  |  |
| **Conclusions** | Page 14, last paragraph. |
| a. Usefulness of the work. | Page 14, last sentence. |
| b. Sustainability. | Page 14, last paragraph. |
| c. Potential for spread to other contexts. | Page 14, last paragraph. |
| d. Implications for practice and for further study in the field. | Page 14, last paragraph |
| e. Suggested next steps. | Page 14, last paragraph, fourth and fifth sentences. |
|  |  |
| **Other information** |  |
| 18. **Funding** - Sources of funding that supported this work. Role, if any, of the funding organisation in the design, implementation, interpretation and reporting. | Page 16, last sub-section on “Funding” covers this. |
|  |  |
|  |  |
|  |  |
|  |  |
| *Ogrinc G, et al. BMJ Qual Saf 2015;0:1–7. doi:10.1136/bmjqs-2015-004411* |  |
| *Downloaded from http://qualitysafety.bmj.com/ on January 2, 2017* |  |
