# Supplementary material for: Improving the healthcare response to domestic violence and abuse in UK primary care: interrupted time series evaluation of a system-level training and support programme
Source: BMC Med. 2020 Mar 5;18:48. doi: 10.1186/s12916-020-1506-3 (PMC7057596; doi:10.1186/s12916-020-1506-3)
Supplement: Supplementary file 3 — Additional file 3. Sample size calculation with code enabling it to be reproduced. [file 12916_2020_1506_MOESM3_ESM.docx]

capture program drop s_its

program define s_its, rclass

version 12

syntax, n(integer) time_points(integer) ///

base_rate_per1000(real) sd_intercept(real) ///

interv_effect(real) time_effect(real) inttime1_effect(real)

drop _all

local base_rate=`base_rate_per1000'/1000

* create n practices, random size with mean 3,000 people,

* with intervention beginning at a random time point

set obs `n'

gen practice_id=_n

gen random_intercept=rnormal(0,`sd_intercept')

gen practice_size=rnormal(3000,1000)

replace practice_size=round(practice_size)

gen lnpractice_size=ln(practice_size)

gen interv_time=1+ceil((`time_points'-1)*runiform())

* expand to give a count for each time-point

expand `time_points'

sort practice_id

by practice_id: gen time=_n

gen interv=(time>=interv_time)

gen inttime1=0

replace inttime1=time-interv_time if interv

* inttime1 is change in effect of intervention over time following intervention

gen rate=`base_rate'*exp(random_intercept)* ///

`interv_effect'^interv*`time_effect'^time*`inttime1_effect'^inttime1

gen count=rpoisson(practice_size*rate)

* then analyse the data with a mixed poisson regression

capture noisily {

xtset practice_id time

xtpoisson count interv time inttime1, re irr offset(lnpractice_size)

return scalar p=2*normal(-abs(_b[interv]/_se[interv]))

}

end

* base rate per 1000 from IRIS trial control group = 0.045

* step change effect of intervention to be detected, IRR = 2

set maxiter 20

simsam s_its n, a(0.05) p(0.90) detect(interv_effect(2.0)) null(interv_effect(1.0)) ///

assuming(base_rate_per1000(0.045) sd_intercept(0.5) time_points(24) ///

time_effect(1) inttime1_effect(1)) pvalue(p) inc(5) prec(0.001) start(100)

* 130 practices needed to detect an IRR of 2 with 90% power at the 5% significance level assuming 24 time points

set maxiter 20

simsam s_its time_points, a(0.05) p(0.90) detect(interv_effect(2.0)) null(interv_effect(1.0)) ///

assuming(base_rate_per1000(0.045) sd_intercept(0.5) n(135) ///

time_effect(1) inttime1_effect(1)) pvalue(p) inc(1) prec(0.001) start(20)

* 23 time points needed to detect an IRR of 2 with 90% power at the 5% significance level assuming 135 practices (11 either side)

set maxiter 20

simsam s_its time_points, a(0.05) p(0.90) detect(interv_effect(2.0)) null(interv_effect(1.0)) ///

assuming(base_rate_per1000(0.045) sd_intercept(0.5) n(180) ///

time_effect(1) inttime1_effect(1)) pvalue(p) inc(1) prec(0.001) start(20)

* 17 time points needed to detect an IRR of 2 with 90% power at the 5% significance level assuming 180 practices
